# Persecutory beliefs predict adherence to epidemiological safety guidelines over time – a longitudinal study

**DOI:** 10.1017/S0033291720002792

**Published:** 2020-07-20

**Authors:** Joachim Kowalski, Łukasz Gawęda

**Affiliations:** Experimental Psychopathology Lab, Institute of Psychology, Polish Academy of Sciences, Warsaw, Poland

**Keywords:** Adherence, compliance, conspiracy, COVID-19, paranoia, psychosis spectrum, SARS-CoV-2

## Background

Recent research studies show negative associations between conspiracy beliefs and adherence to epidemiological safety guidelines (Allington, Duffy, Wessely, Dhavan, & Rubin, 2020; Freeman et al., 2020; Georgiou, Delfabbro, & Balzan, 2020; Kowalski, Marchlewska, Molenda, Górska, & Gawęda, n.d.; Marinthe, Brown, Delouvée, & Jolley, 2020). Recent studies point out to positive associations between coronavirus conspiracy beliefs and paranoia-like beliefs (Freeman et al., 2020) and a potential role of paranoia-like beliefs as originators of coronavirus conspiracy beliefs (Kowalski et al., n.d.). The role of paranoia-like beliefs in lesser adherence to safety guidelines seems partially contradictory with findings linking such beliefs with involvement in safety behaviours (Freeman et al., 2007), as these behaviours could coincide with epidemiological safety measures (e.g. physical isolation, avoiding public places).

In the current study, we aimed at exploring the role of paranoia-like beliefs in predicting changes in adherence to safety guidelines during an epidemic, while controlling for conspiracy beliefs, coronavirus-related anxiety and internal motivation to isolation. These were significant predictors of adherence to safety guidelines in our previous study (Kowalski et al., n.d.).

## Methods

The study was conducted during April and May, with two weeks between measurements; mean 14.67 days (±2.65). Detailed descriptions of governmental guidelines and restrictions are provided in our previous study (Kowalski et al., under review). We obtained *n* = 110 results.

There were 70 (63.6%) women in the sample. In all, 1.8% of participants had primary or vocational education, 29.1% had secondary education, and 69.1% had higher education. In total, 11.8% participants lived in the country, 20.0% in a town below 100k inhabitants, 23.6% in a small city between 100k and 500k inhabitants and 44.5% in a large city above 500k inhabitants.

In this study, we used *Coronavirus related anxiety*, a measure consisting of five items related to the anxiety of self or family members contracting the coronavirus. Higher scores indicate greater levels of anxiety. A measure of *Adherence to safety guidelines* – five items related to official recommendations, like wearing masks in public and self-isolation. Higher scores indicate greater levels of declared compliance with safety measures. *Green Paranoid Thoughts Scale-Revised* (*GPTS-R*) – is a comprehensive measure of paranoia-like beliefs (Freeman et al., 2019). It contains two subscales – Reference and Persecutory beliefs. *Conspiracy beliefs* – measure consisting of 12 items with conspiracy beliefs regarding coronavirus epidemic. Specific items are listed in our previous study (Kowalski et al., n.d.). *Internal motivation to isolation* – single item stating ‘I perceive recommendations to isolate as internally motivated, e.g. to protect my and/or others health’. All measurements had acceptable internal consistency, ranging from *α* = 0.73 to *α* = 0.95.

For statistical analyses, we created a regression model with the difference between T2 and T1 of adherence to safety guidelines as a predicted variable, to reflect change over time in adherence to the said guidelines. We used paranoia-like and conspiracy beliefs, and internal motivation to isolation and coronavirus-related anxiety from T1 as predictors in the created model.

## Results

**Fig. 1.**
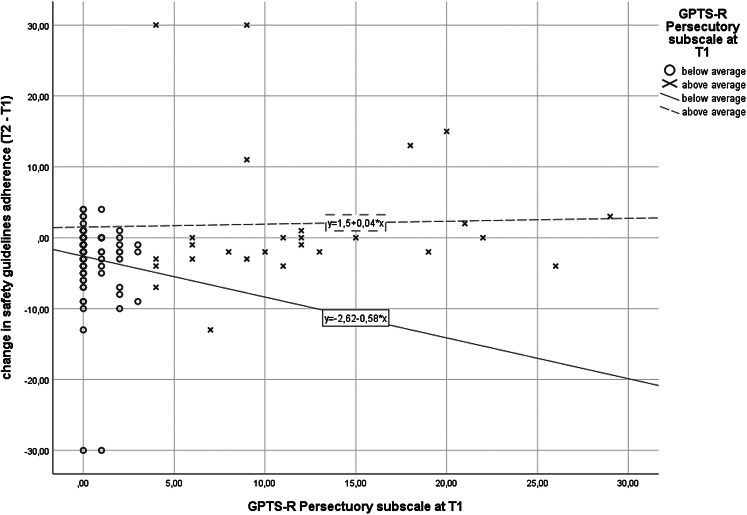
Relation of GPTS-R Persecutory subscale at T1 with change in adherence to safety guidelines (T2–T1) with division of participants into two subgroups based on mean score of GPTS-R Persecutory at T1 for visualization purposes.

Proposed regression model was significant *F*(5,104) = 2.71, *p* = 0.024, adjusted *R*^2^ = 0.07, and revealed that only GPTS-R Persecution was a significant predictor (*β* = 0.44, *p* = 0.005) of change in adherence to guidelines. Details of this relation is presented in Fig. 1, with participants divided into two subgroups based on mean score of GPTS-R Persecution. Internal motivation to self-isolation (*β* = −0.17, *p* = 0.11), GPTS-R Reference (*β* = −0.20, *p* = 0.18), coronavirus conspiracy beliefs (*β* = −0.15, *p* = 0.14) and coronavirus-related anxiety (*β* = 0.13, *p* = 0.19) were not significant in the proposed model.

## Discussion

In this study, we were able to uncover the role of persecutory paranoia-like beliefs in the change of adherence to epidemiological safety guidelines over time. Our results suggest that higher levels of persecutory beliefs are related to continuance of adherence to safety guidelines. This result is in line with accounts linking paranoia-like beliefs to greater engagement in safety behaviours (Freeman et al., 2007), considering that epidemiological safety guidelines may coincide with safety behaviours (i.e. isolation from others, keeping distance). Previous results suggest a positive relation between paranoia-like beliefs, conspiracy beliefs, and lower adherence to safety measures (Freeman et al., 2020; Kowalski et al., n.d.). Our study points out to equivocal relation of paranoia-like beliefs and adherence to epidemiological guidelines and has limitations. A relatively small sample and that our longitudinal sample has skewed demographic characteristics towards larger cities and higher education in relation to the whole T1 sample. This affects the generalizability of our results. Nevertheless, it is the first study to address the role of paranoia-like beliefs in adherence to epidemiological guidelines in a longitudinal design. Further studies are warranted.
